# Integrated pan-cancer analysis revealed therapeutic targets in the ABC transporter protein family

**DOI:** 10.1371/journal.pone.0308585

**Published:** 2025-05-30

**Authors:** Madahiah Bint E Masood, Iqra Shafique, Muhammad Inam Rafique, Ayesha Iman, Ariba Abbasi, Mehak Rafiq, Uzma Habib

**Affiliations:** 1 School of Interdisciplinary Engineering & Sciences (SINES), National University of Sciences & Technology, Islamabad, Pakistan; 2 Department of Biomedical Engineering and Sciences, School of Mechanical & Manufacturing Engineering, National University of Sciences & Technology, Islamabad, Pakistan; Instituto do Cancer do Estado de Sao Paulo / University of Sao Paulo, BRAZIL

## Abstract

Next-generation sequencing technology enables uniform and impartial assessment of cancer diagnoses and prognosis. However, such studies are mostly type-specific, and capturing shared genomic abnormalities responsible for neoplastic transformation and progression is a challenging task. Pan-cancer analysis offers insights into the shared and unique molecular mechanisms driving cancer. We conducted an integrated gene-expression analysis using 10,629 samples from 30 distinct cancer types characterized by The Cancer Genome Atlas (TCGA). A gene co-expression network was constructed and genes overlapping between the selected modules and Differentially Expressed Genes (DEGs) were designated as genes of interest. Following a comprehensive literature review, ATP binding cassette subfamily A member 10 (ABCA10) and ATP binding cassette subfamily B member 5 (ABCB5) were selected as key candidates for downstream analysis due to the absence of systematic pan-cancer analysis of these genes. This study presents a unique contribution as the first comprehensive pan-cancer analysis of ABCA10 and ABCB5, highlighting their roles in tumor biology and clinical outcomes. We employed a variety of bioinformatics tools to explore the role of these genes across different tumors. Our research demonstrated that ABCA10 shows reduced expression, while ABCB5 displays variable expression patterns across tumors, indicating their opposing roles and flexible functions in pan-cancer. In many cancer patients, these expression patterns are correlated with worse survival outcomes. Furthermore, immunotherapy responses and immune infiltration across a variety of tumor types are associated with the expression levels of both ABCA10 and ABCB5. These results imply that ABCA10 and ABCB5 could serve as valuable predictive markers and potential therapeutic targets across various cancers.

## Introduction

Cancer is a formidable enemy that is extremely dangerous for human health and is one of the most important and deadly diseases of our day. The impact of the growing worldwide tumor burden is profound and necessitates quick diagnosis and creative treatment solutions. Regardless of substantial advances in research, Cancer continues to be a leading cause of mortality, with nearly 10 million deaths attributed to it in 2024 [1, 2]. Cancer is an extremely diverse disease, both morphologically and genetically, associated with an assortment of genetic and regulatory anomalies within the cell. Despite decades of scientific progress in cancer treatment clinical benefit from new drugs remains modest in several cases. Therefore, it is crucial to delve into the molecular mechanisms driving cancer. The investigation of prospective biomarkers for cancer diagnosis and treatment outcomes is critical in the field of cancer prevention and control [3–6].

The emergence of Next-generation sequencing (NGS) technology has transformed the expeditious and meticulous identification of genetic anomalies driving cancer. Modern genomic techniques rely on NGS, which can process millions of clonally amplified DNA templates in parallel within a flow cell. This competence has facilitated the exploration of genetic variation, gene expression, epigenetic changes, and microbial diversity, opening up new avenues for research in these areas [7]. The utilization of genome-wide expression data permitted the identification of Differentially Expressed Genes (DEGs) during the course of disease progression. This approach has been crucial for unveiling potential biomarkers for tumor diagnosis, treatment, and prognosis [8, 9]. The weighted gene co-expression network analysis (WGCNA) algorithm is a distinctive biological approach designed to identify highly linked gene modules and important genes using gene expression data [10, 11]. Next-generation sequencing technologies and high-throughput approaches have recently advanced, allowing for more precise and comprehensive examination of global gene expression levels [12]. Nonetheless, there is a challenge: these analyses tend to concentrate on specific tumor types, whereas molecular research reveals that tumors from various organs may share common traits, while tumors originating from the same organ could exhibit significant variations [13–16]. NGS, pan-cancer model systems, and initiatives like The Cancer Genome Atlas (TCGA) are tools used in pan-cancer analysis to identify genes and genetic anomalies that are commonly altered across various cancer types, irrespective of their origin [17].

In our research endeavors, we have leveraged RNAseq data and clinical records for comprehensive pan-cancer investigations. The datasets were predominantly sourced from TCGA [18–20], a repository renowned for its extensive collection of genetic sequences from over 30 malignancies. Notably, each cancer subtype within the TCGA database encompasses data from as many as 500 tumors, thereby offering a rich and diverse dataset for our studies. By contrasting gene expression patterns between tumor (T) and adjacent non-tumor (NT) tissues, we identified a number of DEGs. These DEGs were subjected to gene set enrichment analysis to unveil enriched pathways and gene sets. Simultaneously, through WGCNA analysis, we identified key co-expression modules. Genes shared between the DEGs and WGCNA lists were designated as genes of interest, with ATP binding cassette subfamily A member 10 (ABCA10) and ATP binding cassette subfamily B member 5 (ABCB5) selected as key candidates. Furthermore, their correlation with gene expression levels, cancer cell sensitivity, and immune response was evaluated across multiple cancer types. Our in-depth bioinformatics exploration highlights the pivotal roles of ABCA10 and ABCB5 genes across a spectrum of cancers, offering insights into potential cancer biomarkers.

## Materials and methods

### Ethics statement

This study utilized publicly available, pre-existing, de-identified TCGA (https://cancergenome.nih.gov/) datasets. It did not involve any additional human participation beyond the original TCGA data collection, and thus did not require Institutional Review Board (IRB) or ethics committee approval. All collected data were fully anonymized before access, and informed consent was not needed.

### Sample retrieval and assessment

**Fig 1:**
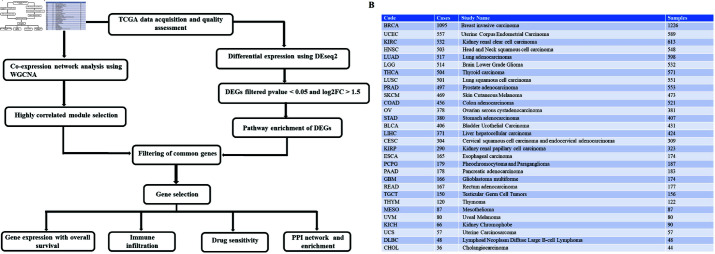
Methodology and datasets used in this study. (**A**) The flowchart of methodology. (**B**) Cancer types and the RNASeq sample count from TCGA used in presented work.

The overall workflow of our study is illustrated in Fig 1A. We have used primary, recurrent, metastatic, additional primary and additional metastatic tumor samples of 30 different types along with their adjacent normal tissue samples (10629 samples altogether). RNA-seq raw count data for 60,660 Ensembl genes representing 30 cancer types were downloaded. The R/Bioconductor package TCGAbiolinks [21]was used to retrieve raw count data via the Genomic Data Commons (GDC) cancer data repository. Fig 1B shows a detailed description of the cancer types used in this study.

### Data preparation

Expression matrices were extracted using the assay() function, which retrieves expression data from SummarizedExperiment objects. The extracted matrices were exported as individual CSV files. The full_join() function was used to merge them into a single dataset. The final combined dataset, containing RNA counts for all samples, was exported as a single CSV file for downstream analysis.

### Filtering of low-read count genes

Before conducting differential expression analysis, we filtered out genes with ≤10 raw counts across all samples. This filtering step was essential to eliminate genes with low expression that could introduce noise and reduce the statistical power of the analysis. After filtering, a total of 58,137 genes remained, which were used for differential expression analysis.

### Screening for DEGs and analysis of pathway enrichment

The R/Bioconductor package DESeq2 [22] was utilized for count normalization, batch effect correction, and the identification of differentially expressed genes, ensuring robust and reliable analysis. The DESeq2 package employs the median-of-ratios method to normalize size factors, correcting for sequencing depth and RNA composition differences across samples. This method calculates size factors based on the median ratio of gene counts to a pseudo-reference sample, which is formed from the geometric mean of counts across all samples. Furthermore, DESeq2 adjusts for batch effects and other technical variations, ensuring that gene expression levels are comparable and DEGs are accurately identified. DEGs were identified by contrasting the transcript levels between malignant and non-malignant samples. The Benjamini–Hochberg approach was used to decrease the false discovery rate and adjust P-values. Subsequently, the signiï¬#129;cant DEGs filtered at thresholds of (*padj*<0.05 and $|\log_{2}(\text{fold-change})|>1.5$) were used for pathway enrichment analysis. Studies have demonstrated that combining fold-change and p-value thresholds provides biologically meaningful gene sets and enhances consistency across platforms [23, 24]. The gene set enrichment analysis tool (GSEA) from the Broad Institute (https://www.gsea-msigdb.org/) was used for enrichment analysis of DEGs. The predefined molecular signature database MSigDB, hallmark (H) gene set (version 7.1) was set as reference gene set for pathway enrichment [25]. The pre-ranked GSEA module was employed with 1000 permutations. Enrichment significance was determined with a False Discovery Rate (FDR) of 25%. Enriched genes within each pathway are displayed through exact images, representing their corresponding p-value along with normalized enrichment score (NES) and false discovery rate (FDR).

### Gene co-expression network construction and detection of tumor-linked modules

R software package “WGCNA” [10] was employed to reconstruct the co-expression network of cancer genes. We begin by validating the quality of genes and samples, followed by the construction of a Pearson correlation matrix. The formula $a_{mn}=|c_{mn}{|}^{\beta }$ (where *c*_*mn*_ represents the Pearson correlation amongst genes, *a*_*mn*_ denotes the adjacency between genes, and β parameter adjusts the correlation strength) was applied to construct the weighted adjacency matrix.

β, the soft threshold power, was chosen on the basis of standard scale-free network criteria. Next, the adjacency matrix served as the foundation for a topological overlap matrix (TOM) [26] and genes were structured into hierarchical clusters to detect groups of co-expressed genes. The minimum module size of 50 was selected for the gene cluster tree, and a cutting threshold of 0.25 was applied to merge similar modules in the modular dendrogram.

### Development of module-trait correlations

Correlation analysis was employed to identify gene modules significantly linked to primary, recurrent and metastatic tumor states. Our analysis focused on modules that exhibited strong associations with specific tumor states, designating them as modules of interest.

### Identification and selection of genes of interest

Using the open source “Venny” v 2.1 software (https://bioinfogp.cnb.csic.es/tools/venny/), a Venn diagram was initially generated to compare module genes with DEGs. Genes that overlap between selected modules and DEGs were designated as genes of interest. Following an exhaustive literature search, two genes, ABCA10 and ABCB5, were chosen for further investigation.

### Gene expression analysis

To explore the expression of selected genes across different tumors, the Gene_DE framework from TIMER 2.0 (http://timer.cistrome.org/)database was employed [27]. GEPIA2 (http://gepia2.cancer-pku.cn) was employed to analyze the expression levels of ABCA10 and ABCB5 across different stages of tumors available in the TCGA database. databases [28]. This integration enables comprehensive analysis of gene expression across both cancer and normal tissue samples. Additionally, GEPIA2 and GENT2 [29] (http://gent2.appex.kr) were utilized to validate the expression levels of ABCA10 and ABCB5 in independent external datasets. GEPIA2 integrates data from TCGA and GTEx, offering independently processed data that provides a complementary perspective and enables a more robust assessment of gene expression patterns. GENT2, which focuses on microarray-based gene expression data from various public datasets, further complements this validation by broadening the data types used for analysis.

### Assessment of survival prognosis

Kaplan-Meier plotter (https://kmplot.com/analysis) was utilized to assess the impact of ABCA10 and ABCB5 expression on patient outcomes across cancers, primarily utilizing data from TCGA databases, and evaluates the influence of over 54,000 genes on survival across 21 different cancer typesv [30]. Patient cohorts were categorized using the ‘Auto Select Best Cutoff’ method, that calculates all probable cut-off points in the range of lower and upper quantiles by default and picks the optimal limit as the cutoff.

### Assessment of immune cell infiltration

The Gene module from the TIMER database was employed to examine the correlation amongst gene transcript levels and immune cell infiltration across TCGA tumors. This analysis involved various subtypes of immune cells, encompassing B cells, CD4+ T cells, CD8+ T cells, macrophages, dendritic cells, and cancer-associated fibroblasts.

### Enrichment analysis and protein–protein interaction (PPI) networks

The Search Tool for the Retrieval of Interacting Genes (STRING) data repository [31] was employed to generate the interaction network of genes associated with ABCA10 and ABCB5. ABCA10 and ABCB5 were submitted to the Multiple Proteins search bar with a confidence score cut-off set at 0.4. Genes that interact with ABCA10 and ABCB5 were further utilized for enrichment analysis. The Kyoto Encyclopedia of Genes and Genomes (KEGG) pathway (www.kegg.jp/kegg/kegg1.html) [32] enrichment of interacting genes, was conducted by Database for Annotation, Visualization and Integrated Discovery (DAVID) (https://david.ncifcrf.gov) database. Eventually, Cytoscape software was used for visualization of the results.

### Drug sensitivity analysis

To investigate the relationship between ABCA10 and ABCB5 gene expression and drug sensitivity, we utilized CellMiner (https://discover.nci.nih.gov/cellminer/), a web-based tool offering data and pharmacological insights on the NCI-60 cancer cell lines, along with CellMinerCDB (https://discover.nci.nih.gov/rsconnect/cellminercdb/) [33–36] for data mining and visualization. Additionally, the Connectivity Map (CMap) (https://clue.io/) was employed as a supplementary resource for drug sensitivity predictions [37].

### Statistical evaluation

Statistical analyses were performed using R software, where the Wald test in DESeq2 was applied to evaluate differential expression among tumor and normal samples. The TIMER database used the Wilcoxon test for tumor versus normal expression comparisons, while GEPIA2 and GENT2 applied Student t-test for pairwise comparisons of gene expression. Kaplan-Meier survival curves were analyzed using the log-rank test to calculate hazard ratios (HR) and associated p-values. Correlations between gene expression and immune infiltration were assessed using Spearman correlation in TIMER. Drug sensitivity correlations were evaluated using Pearson correlation coefficient to examine the relationship between gene expression levels and drug activity in NCI-60 cell lines. P values below 0.05 were regarded as statistically significant.

## Results

### Data preprocessing and identification of DEGs

**Fig 2:**
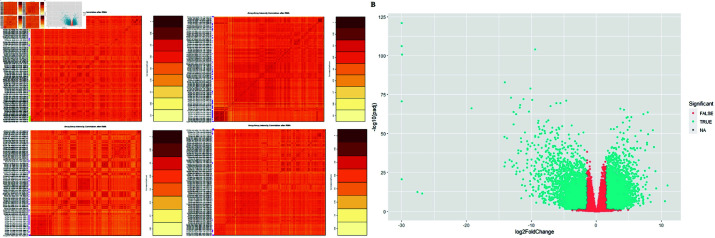
AAIC and DEGs visualization. (A) Array-array intensity correlation (AAIC). (B) Volcano plot of DEGs.

Data preprocessing was done by constructing Array Array Intensity correlation (AAIC) which defines a square symmetric matrix of spearman correlation among samples to identify problematic arrays [38]. AAIC was visualized (Fig 2A), the colors represent the strength of the correlation between the samples. The correlation cut-off equal to 0.6 is used to determine the outliers, a higher correlation is shown in a dark color and lower correlation in a light color. The DEseq2 R software package was employed to identify common DEGs in tumor vs normal tissues across 30 different cancer types from TCGA. The volcano plot (Fig 2B) was used to illustrate DEGs, there were 2955 DEGs including 1381 upregulated genes and 1574 downregulated genes (S1 Table)

### Gene set enrichment analysis (GSEA)

**Fig 3:**
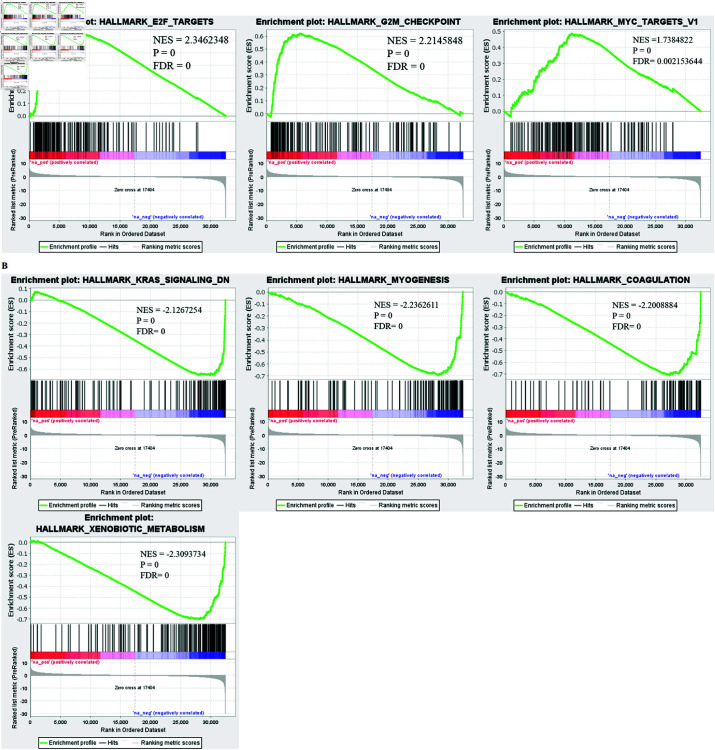
Gene set enrichment analysis of DEGs. (A) Displays the pathways enriched in upregulated DEGs. (B) Illustrates the pathways enriched in downregulated DEGs. Normalized enrichment score (NES), false discovery rate (FDR).

We used DEGs to perform gene set enrichment analysis (GSEA) for cancer hallmark pathways in order to evaluate the oncogenic pathways linked to cancer. GSEA reveals that upregulated DEGs were significantly enriched in E2F targets, G2M checkpoint and MYC targets (Fig 3A). Meanwhile, Fig 3B depicts that downregulated DEGs were enriched in xenobiotic metabolism suggesting the hyperactive metabolism response of cancer cells, besides other metabolic pathways including myogenesis, coagulation and KRAS signaling.

### Co-expression network analysis (WGCNA)

**Fig 4:**
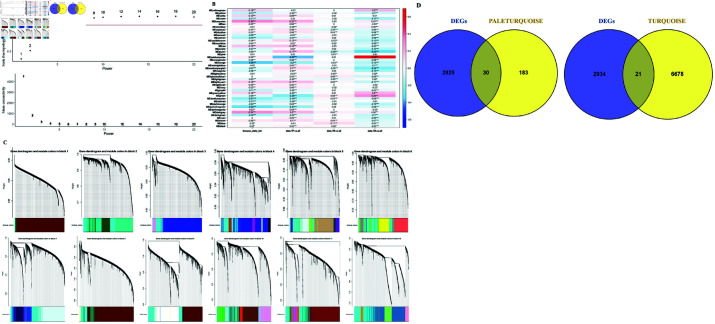
WGCNA and identification of genes of interest. (**A**) Evaluation of network topology performed across a range of soft-threshold powers. (**B**) Module–trait correlation heatmap (* *P*<0.05; ** *P*<0.01; *** *P*<0.001). (**C**) Gene clustering dendrograms (D) Venn diagram of the DEGs and modules.

We employed the WGCNA R software package to categorize genes with related expression patterns into distinct groups, genes within a group demonstrate a comparable pattern of co-expression. Through a series of quality assessments for the gene expression matrix, a soft threshold of 8 was picked to construct and validate the scale-free network (Fig 4A). 43 modules were obtained by setting minimal module size as 50 genes, max block size 4000 and cut height as 0.25 (Fig 4C) to merge similar modules. The heatmap of module-trait correlations (Fig 4B) revealed that the turquoise module (cor = 0.33, *P*<0.001) was most correlated with tumor state, whereas the paleturquoise module (cor = 0.69, *P*<0.001) was most correlated with metastatic tumor. The turquoise module included 6699 genes, while the paleturquoise module had 213 genes. As demonstrated in Fig 4D, DEGs and the turquoise module shared 21 genes, while the paleturquoise module shared 30. An exhaustive literature search led to the selection of two genes, ABCA10 and ABCB5 for downstream analysis.

### ABCA10 and ABCB5 expression levels across TCGA cancers

**Fig 5:**
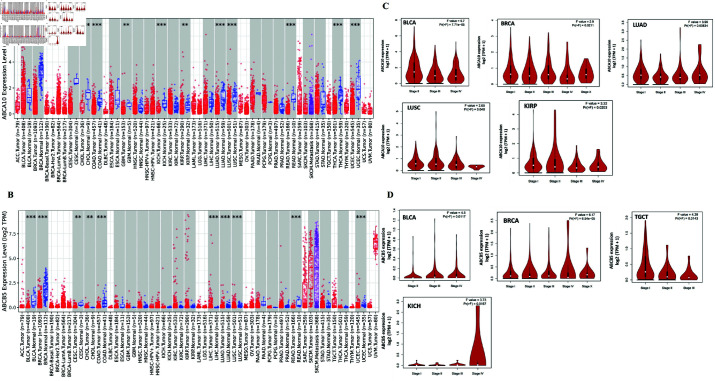
ABCA10 and ABCB5 expression levels across various cancers and their stages.

To assess the expression levels of ABCA10 and ABCB5, the TIMER database has been employed. Fig 5A illustrates that ABCA10 expression in tumors was notably lower compared to corresponding normal tissues, such as BRCA, CHOL, COAD, KICH, LUAD, LUSC, READ, THCA, UCEC *P*<0.001, CESC, GBM, KIRP *P*<0.01. Similarly, ABCB5 demonstrated reduced expression in BLCA, BRCA, COAD, READ *P*<0.001 and CESC *P*<0.01. Additionally, ABCB5 expression was markedly elevated in LIHC, LUAD, UCEC, LUSC *P*<0.001 and CHOL *P*<0.01 then in normal samples (Fig 5B). Furthermore, based on the ’stage plot’ feature of GEPIA2, we observed that mRNA expression of ABCA10 in BLCA, BRCA, LUAD, LUSC and KIRP *P*<0.05 was correlated with tumour stage (Fig 5C), although no correlation was detected in other malignancies. Meanwhile, ABCB5 expression in BLCA, BRCA, KICH and TGCT *P*<0.05 exhibited a correlation with tumor stages, unlike other tumors, as shown in Fig 5D. Additionally ABCA10 shows significant downregulation across cancers when analyzed using GEPIA2, as data for this gene was unavailable in GENT2. The differential expression of ABCB5 was further supported by GENT2, underscoring its multifaceted roles in pan-cancer contexts (S1 fig).

### Prognostic value of ABCA10 and ABCB5 across cancers

**Fig 6:**
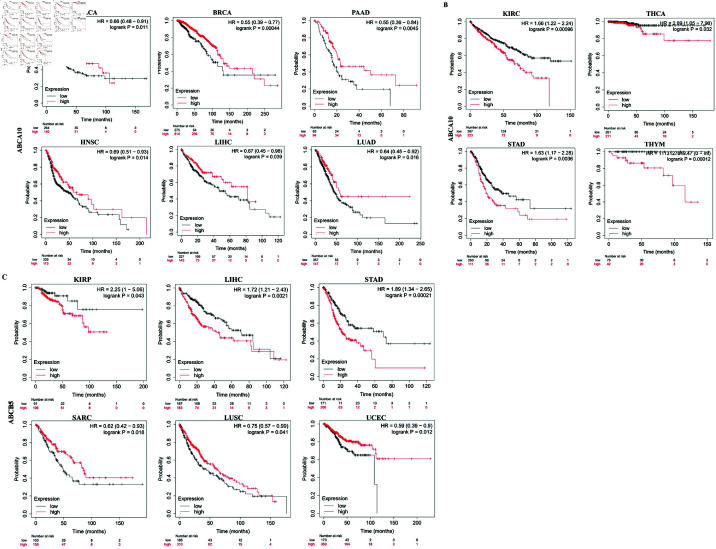
Comparison of low and high expression levels of ABCA10 and ABCB5 across various tumors using survival analysis.

The correlation between ABCA10 and ABCB5 expression levels and patient prognosis was explored through the Kaplan–Meier Plotter tool. Notably, significant correlation was observed between ABCA10 expression and clinical outcome in 10 cancer types, and between ABCB5 expression and prognosis in 6 cancer types. Additionally, the prognosis of STAD and LIHC was linked to the expression of both ABCA10 and ABCB5. However, the two genes play different roles in LIHC and similar roles in STAD. Based on the median expression value, patients were stratified into higher and lower expression groups. The study also revealed that the survival rate was lower in the low-ABCA10 group relative to high-ABCA10 group in BLCA, BRCA, PAAD, HNSC, LIHC and LUAD (Fig 6A). Conversely, (Fig 6B) a shorter overall survival was associated with elevated expression of ABCA10 in KIRC, THCA, STAD, and THYM. However, low ABCB5 expression was linked with better survival outcomes in KIRP, LIHC, STAD, and showed worse survival in SARC, LUSC, and UCEC (Fig 6C).

### Enrichment analysis of genes correlated with ABCA10 and ABCB5

**Fig 7:**
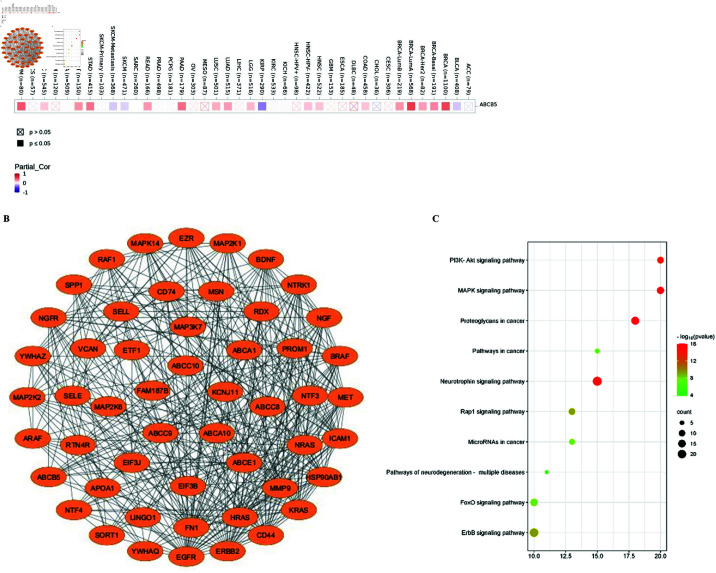
Enrichment of genes correlated with ABCA10 and ABCB5.

Fig 7A demonstrates positive correlation between the expression levels of ABCA10 and ABCB5. Thus, we utilized the STRING web tool to screen for proteins that interact with ABCA10 and ABCB5, in order to investigate their molecular mechanism in carcinogenesis. The interaction network of 52 proteins was established as shown in Fig 7B. The gene set was used to perform KEGG pathway enrichment analysis using the DAVID online tool. According to Fig 7C, the most highly enriched KEGG pathways including “PI3K-Akt signaling pathway”, “MAPK signaling pathway”, “Proteoglycans in cancer”, “Rap1 signaling pathway”, “FoxO signaling Pathway” and “ERBB signaling pathway” could potentially contribute to regulating the impacts of ABCA10 and ABCB5 in tumor development.

### Correlation of immune cell infiltration with gene expression

**Fig 8:**
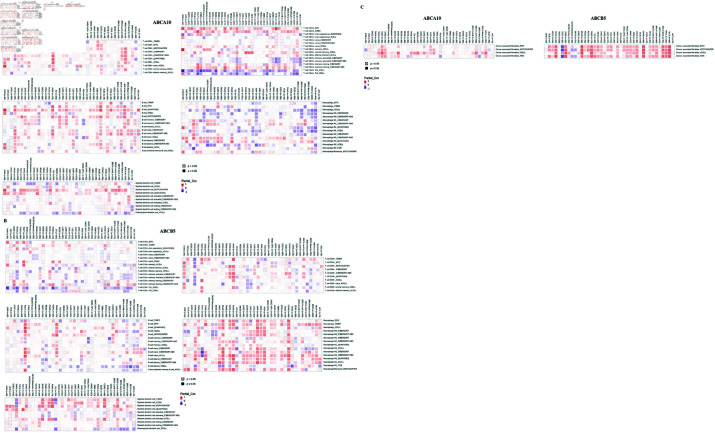
Correlation of ABCA10 and ABCB5 expression with the extent of immune cell infiltration.

Next, we investigated the possibility of a relationship between immune infiltration level and gene expression in distinct cancer types through various immunological databases. The outcomes presented a strong relationship between ABCA10, ABCB5 and different levels of immune cell infiltration. Significant positive correlations were observed between the expression of ABCA10 and the abundance of T cells, B cells and DCs, whereas negative correlation was observed with macrophages (Fig 8A). Conversely, ABCB5 expression exhibited a positive correlation with DCs and macrophages across most cancer types. Additionally, ABCB5 expression exhibited a positive correlation with B cells in TGCT and PAAD. While T cells displayed a negative correlation with ABCB5 expression in several cancer types, exceptions were observed in BRCA, PCPG, PAAD, and UVM (Fig 8B). Furthermore, ABCA10 expression in BRCA, STAD, and TGCT was significantly positively correlated with cancer-associated fibroblasts, which play a role in tumor development [39]. Similarly, a positive correlation between ABCB5 expression and cancer-associated fibroblasts was observed in most tumors, except for TGCT (Fig 8C).

### Evaluation of drug sensitivity

**Fig 9:**
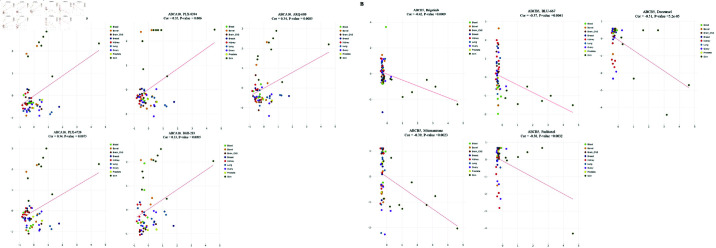
The relationship of ABCA10 and ABCB5 gene expression with drug sensitivity.

We evaluated the relationship between the activity of drug compounds and expression levels of ABCA10 and ABCB5 Fig 9A demonstrates that elevated ABCA10 expression correlates with increased drug sensitivity to PLX-4720, PLX-8394, BGB-283, ARQ-680 and Vemurafenib. However, ABCB5 displayed negative association with antitumor drugs (Fig 9B) such as BLU-667, Paclitaxel, Docetaxel, Mitoxantrone and Brigatinib proposing potentials to drug resistance. Using the median values of ABCA10 and ABCB5 gene expression, tumor cell lines were classified into groups with elevated and reduced expression levels, to obtain DEGs. Top 150 genes were used to retrieve corresponding compounds (S2 Table). As seen in Table 1 we observed that similar entities were identified for ABCA10 and ABCB5 (highlighted with a yellow box and red font for prominent presentation) depicting |CMap Score|<90, very low correlation [40]. This discrepancy between the results from the two databases could be attributed to the differences in the number of chemicals included in each database.

**Table 1:** CMap analysis results for ABCA10 and ABCB5 genes (CMap score > 0 means positive correlation, while CMap score < 0 means negative correlation).

ABCA10		ABCB5	
Compound	CMap Score	Compound	CMap Score
PLX-4720	-65.96	Docetaxel	-44.47
Vemurafenib	35.03	Paclitaxel	14.29
		Mitoxantrone	-58.24

## Discussion

Despite decades of scientific progress in cancer treatments, it remains the second significant reason for death worldwide. This implies that the complex mechanism involved in tumorigenesis has not been fully elucidated so far. Primarily due to the fact that cancer cells exhibit extensive heterogeneity, encompassing both morphological and genetic alterations. Pan-cancer analysis not only offers valuable insights into the common and distinct molecular mechanisms that initiate cancer, regardless of tumor origin, but it also has enormous potential for the discovery of effective diagnostic biomarkers and prognosis predictors that are common across all cancer types.

Initially, 2955 shared DEGs between malignant and non-malignant tissues were identified using DEseq2 R package. we performed gene set enrichment analysis to further determine the role of DEGs with underlying oncogenic pathways. Upregulated DEGs were predominantly enriched in cell proliferation associated pathways, including E2F targets, G2M checkpoint and MYC targets. It has been reported in numerous studies [41–43]that increased cell proliferation is linked with poor survival. E2F participates in numerous physiological and pathological processes, such as the tumor cell cycle [44], DNA damage response [45], cell proliferation, differentiation, and cell death [46]. It has emerged as one of the main transcriptional modulator of cell cycle-regulated gene activity. Additionally, GSEA demonstrated the enrichment of downregulated DEGs in KRAS signaling, coagulation and myogenesis. The KRAS mutation pathway, which is frequently activated in tumor cells, accounts for enhanced apoptosis inhibition, migration, and proliferation [47–49]. Enrichment analysis indicated that xenobiotic metabolism was also among the significantly enriched pathways. The metabolic inclinations that cancer cells developed were advantageous for their nutritional needs during growth and multiplication, confirming the significant impact of reprogramming cell metabolism on carcinogenesis [50].

WGCNA has been used extensively to uncover biomarkers that predict disease progression. WGCNA is a useful algorithm for discovering modules of co-expressed genes, module is a collection of genes with matching expression patterns across various biological mechanisms or sample types [10, 51]. After module identification, the association among gene modules and tumor state (primary, recurring, and metastatic) was calculated. 43 modules were identified in present study, correlation analysis reveals that turquoise and paleturquoise modules are the modules of interest, exhibiting strong relationships with different tumor states. Subsequently, we identified genes that were common between DEGs and genes in selected modules. This process ensures that every gene exhibits the greatest degree of differential expression accompanied by highest correlation with tumor state. Ultimately, ABCA10 and ABCB5 were selected for further analysis.

ABC transporters have been extensively investigated in the context of cancer, where they play pivotal roles in immune regulation and treatment resistance. Their functions are well-documented, particularly in the context of multidrug resistance, where these proteins actively pump chemotherapeutic agents out of cancer cells, diminishing drug efficacy. Comprehensive studies have detailed the structural and functional complexity of ABC transporters and their involvement in various biological processes. However, research has predominantly focused on well-known members, such as multidrug resistance protein 1 (MDR1, also known as ATP-binding cassette subfamily B member 1, ABCB1), breast cancer resistance protein (BCRP, also known as ATP-binding cassette subfamily G member 2, ABCG2), and multidrug resistance-associated protein 1 (MRP1, also known as ATP-binding cassette subfamily C member 1, ABCC1) [52–54] leaving other transporters relatively underexplored. While emerging research is beginning to uncover roles of ABC transporters apart from drug efflux, including their influence on tumor biology and immune interactions, these studies typically focus on a limited number of cancer types. Consequently, a comprehensive understanding of the broader roles of less-studied transporters, such as ABCA10 and ABCB5, remains an important area for further exploration. We utilized an innovative bioinformatics approach to bridge this significant gap by simultaneously examining ABCA10 and ABCB5 across multiple cancer types. Effectively integrating gene expression data, patient survival outcomes, immune cell infiltration patterns, and drug sensitivity profiles, our study goes beyond traditional single-cancer research. This multidimensional analysis reveals that ABCA10 and ABCB5 play distinct and opposing roles in tumor biology, providing valuable insights into their potential as prognostic biomarkers and therapeutic targets. To the best of our knowledge, this study is the first to conduct a comprehensive and systematic pan-cancer analysis of ABCA10 and ABCB5, exploring their correlations with immune cell infiltration and drug responses across multiple cancer types utilizing advanced multiomics tools.

The TIMER and GEPIA2 databases were used to analyze the gene expression levels of ABCA10 and ABCB5 in different tumors and their stages. Our analysis demonstrated that the expression of ABCA10 was reduced in most tumors, including LUAD and BRCA, supporting its function as a potential tumor suppressor. Whereas ABCB5 shows variable expression across tumors, reflecting its context-dependent roles. Many proteins and genes exhibit such roles influenced by tumor stage, cell type, microenvironment, and genetic context [55]. Consistent with our findings, Yang *et al*. reported a notable underexpression of ABCA10 in LUAD, further associating this downregulation with TP53 mutations in LUAD patients [56]. Similarly, Chu *et al*. documented the reduced expression of ABCA10 across distinct breast cancer subgroups [57]. Additionally, Li *et al*. observed pronounced downregulation of ABCA10 at transcription and translation levels in ovarian cancer tissues in comparison to normal controls. Their study illustrated that ABCA10 facilitates lipid metabolism reprogramming by promoting mitochondrial cholesterol efflux, thereby increasing the sensitivity of ovarian cancer cells to cisplatin (DDP). They also identified transcription factor 21 (TCF21) as a direct transcriptional regulator of ABCA10, providing valuable insights into the molecular mechanisms of ovarian cancer resistance to DDP and proposing potential avenues for therapeutic intervention [58]. Furthermore, our results align with prior research, including the study conducted by Dvorak *et al*. [59] and Hlavac *et al*. [60], that highlighted the prognostic importance of ABC transporter expression. Specifically, our findings demonstrated that reduced ABCA10 expression level is linked with worse survival outcomes in several cancers. This aligns with the broader evidence suggesting that downregulation of certain ABC transporters may adversely impact survival outcomes, underscoring the potential of ABCA10 as a prognostic biomarker.

Unlike ABCA10, multiple studies [61–64] have demonstrated the overexpression of ABCB5 across various cancer types, highlighting its significant role in promoting chemoresistance. Our analysis corroborates these findings, showing ABCB5 overexpression in LIHC, LUAD, UCEC, and LUSC. A recent study [65] further revealed that in oral squamous cell carcinoma (OSCC) and oral cancer stem cells (CSCs), elevated levels of Long Intergenic Non-Protein Coding RNA 963 (LINC00963) drive malignant progression and drug resistance through the regulation of ABCB5. The molecular importance of ABCB5 in promoting cancer stemness and chemoresistance is underscored by their findings, which suggest that targeting LINC00963 could sensitize resistant cancer cells and improve therapeutic outcomes in OSCC patients. Another study by Li and Hou [66] demonstrated that ABCB5 plays an important role in the progression of pancreatic cancer. Specifically, their research highlighted that microRNA-4282 (miR-4282) limits the metastatic potential of pancreatic cancer cells by reducing ABCB5 expression. The study found that lower levels of miR-4282 were correlated with higher ABCB5 expression, advanced metastasis, and worse survival in patients with pancreatic cancer. Furthermore, elevated ABCB5 expression has been linked to tumor progression in melanoma, indicating an association with increased tumor aggressiveness [67].

Our analysis indicates that higher expression of ABCB5 is correlated with worse survival outcomes in tumor patients. The clinical significance of ABCB5 has been extensively documented, showing strong correlations between its expression and poorer patient outcomes, such as increased tumor progression and reduced overall survival in colorectal and oral squamous cell carcinomas. Studies [68]have also demonstrated that reducing ABCB5 expression sensitizes cancer cells to chemotherapeutic agents. These findings, together with our results, highlight the pivotal role of ABCB5 in tumor biology and emphasize its promise as a drug target to combat treatment resistance.

ABC transporters serve as modulatory tumor suppressors, facilitating cell death through the mitochondrial network. Tumor suppression is mediated by the modulation of intracellular AKT signaling [69]consistent with our results. Mitochondria play a pivotal role in regulating ABC transport proteins, their absence leads to an increase in cytotoxic and helper T cells, as well as the production of Interferon-gamma (IFN-γ) secreted by these cells [70].

ABC transporters perform crucial functions in tumor progression and resistance to treatment via their influence on the tumor immune microenvironment (TIME), cytokine regulation and cellular metabolism. ABCB5 is overexpressed in tumor stem cells and is correlated with chemoresistance and tumor recurrence. In melanoma, it is known to drive the production of Interleukin-1 beta (IL-1β), which sustains chemotherapy-resistant cells through an IL-1β/Interleukin-8 (IL-8)/C-X-C motif Chemokine Receptor 1 (CXCR1) signaling circuit, establishing a response that reinforces tumor growth and survival. Additionally, ABCB5 modulates immune cell behavior in the TIME, influencing the differentiation and migration of macrophages and T-cells, thereby creating an environment that favors tumor growth and resistance to immunotherapy [71–75].

ABCA10 appears to exert tumor-suppressive effects through its role in mitochondrial cholesterol efflux and modulation of lipid metabolism, which are essential for maintaining cellular homeostasis. Dysregulation of these processes, as observed in ovarian and lung cancers, may contribute to reduced apoptosis and enhanced tumor growth. Additionally, our findings align with studies suggesting that ABCA10 downregulation impacts AKT signaling pathways, potentially inhibiting cell death and promoting survival. Conversely, ABCB5 demonstrates a strong association with chemoresistance and immune suppression, driven by its role in regulating cancer stemness and IL-1β/IL-8/CXCR1 signaling loops. These mechanisms create a tumor microenvironment that fosters drug resistance and immune evasion, consistent with our findings of its positive correlation with macrophage infiltration. The distinct and opposing roles of ABCA10 and ABCB5 may reflect a complex interplay between tumor suppressive and oncogenic forces, necessitating further research to delineate their context-specific functions.

Overexpression of ABC transporters has been implicated not only in mediating multidrug resistance (MDR) due to increased drug efflux: but also in reducing the efficacy of tumor immunotherapy. Dysregulation of these transporters affects immune cell development, differentiation and migration, influencing immune responses at sites of inflammation. Resistance to immunotherapy, including inhibitors of immune checkpoint that target Programmed Cell Death Protein 1 (PD-1) and Programmed Death-Ligand 1 (PD-L1), is a major clinical challenge. For instance, Transporter Associated with Antigen Processing 1 (TAP1)/ATP-Binding Cassette Subfamily B Member 2 (ABCB2) deficiency has been linked to increased CD8+ T cells and decreased tumor-associated neutrophils and CD4+ regulatory T cells, enhancing anti-PD-1 responses. Conversely, tumors overexpressing TAP1/ABCB2 exhibited resistance to anti-PD-1 therapy. These findings underscore the critical role of ABC transporters in mediating immune resistance and shaping the tumor immune microenvironment [76, 77].

The expression of ABCA10 was positively correlated, while ABCB5 was negatively correlated with immune infiltration levels in multiple tumors. With the exception of macrophages, which showed a negative correlation, ABCA10 exhibited positive associations with the majority of immune cells. In contrast, a positive association was observed between macrophages and ABCB5. These findings imply that ABCA10 and ABCB5 have different immune-modulatory functions in shaping the tumor microenvironment. The tumor-suppressive potential of ABCA10 is further supported by its positive correlation with immune cells. On the other hand, the positive association between ABCB5 and macrophages aligns with its role in creating an immunosuppressive milieu that contributes to chemoresistance.

Although immunocheckpoint inhibitors have demonstrated remarkable success in treating a variety of malignancies, dysregulated expression of the ABC transporter may lessen their effectiveness and make tumors less susceptible to immunotherapy. This underscores the need for further research into the interaction between ABC transporters and immune resistance mechanisms to improve treatment outcomes [78–80].

Furthermore, the apparent correlation of ABCA10 and ABCB5 with cancer-associated fibroblasts across tumors highlights their broader influence on TIME. Cancer-associated fibroblasts have important roles in tumor growth and resistance mechanisms, including immune evasion and therapeutic resistance. These correlations present important insights into the functional roles of these transporters and their viability as drug targets to address therapy resistance in cancer.

ABC transporters are essential for regulating immunological responses and sustaining cellular homeostasis, according to recent research. Such as, ABCC5 has been associated with immune infiltration and immune cell differentiation in hepatocellular carcinoma, while ABCA1 deletion has been linked to increased inflammatory cell death. Likewise, ABCA3 has been associated with modulating surfactant metabolism and lipid homeostasis during lung inflammation, which in turn affects inflammatory processes. These findings illustrate diverse and important roles that ABC transporters play in immune modulation and inflammatory responses [81–83].

Our research advances our knowledge of the unique and opposing functions of ABCA10 and ABCB5 in drug resistance and immune regulation. In addition to many in silico studies in the literature, data from TCGA has provided valuable insights into cancer biology [84–86]. Future research ought to delve into the molecular processes that underpin these activities in order to uncover new approaches to target these transporters in various cancers. Studies should also look into the processes underlying cancer-specific changes and confirm the context-dependent expression of ABCB5 in different cohorts. To clarify the molecular mechanisms connecting ABCA10 and ABCB5 to immunological responses, mechanistic research is also desired.

Despite highlighting the important roles of ABCA10 and ABCB5 in carcinogenesis and patient survival, our bioinformatics-based research has some limitations , particularly the lack of in vitro and in vivo experiments. Potential biases resulting from differences in data collection and processing methods among databases, including those generated primarily from TCGA, underscore the need for more standardized and comprehensive datasets to provide robust and reliable results. Future in vivo and in vitro studies are planned to corroborate our findings and acquire a better understanding of the functional roles of ABC transporters, such as ABCA10 and ABCB5. Moreover, future research may expand its focus to include other ABC transporters, providing a more thorough understanding of their roles in cancer progression and treatment resistance.

The first in-depth pan-cancer analysis of ABCA10 and ABCB5 is presented in this study, offering valuable insights into their diverse roles in tumor biology. According to our research, ABCA10 has decreased expression, while ABCB5 exhibits varying expression patterns among tumors, indicating their different and possibly opposing functions in some malignancies. The differential expression of these genes reveals their possible involvement in different aspects of tumor biology, suggesting that ABCA10 and ABCB5 may play distinct and opposing roles in cancer development and progression. These results provide a foundation for further research investigating their potential as therapeutic targets or biomarkers.

## Supporting information

S1 Table Differentially expressed genes

S2 Table CMap analysis results for ABCA10 and ABCB5 genes.

S1 File 52 Proteins that interact with ABCA10 and ABCB5.

S2 File Protein interaction network.

S3 File Paleturquoise module genes.

S4 File Turquoise module genes.

S1 Fig Validation of expression levels of ABCA10 and ABCB5.
