# Supplementary figures and images for: Integrated pan-cancer analysis revealed therapeutic targets in the ABC transporter protein family
Source: PLoS One. 2025 May 30;20(5):e0308585. doi: 10.1371/journal.pone.0308585 (PMC12124511; doi:10.1371/journal.pone.0308585)

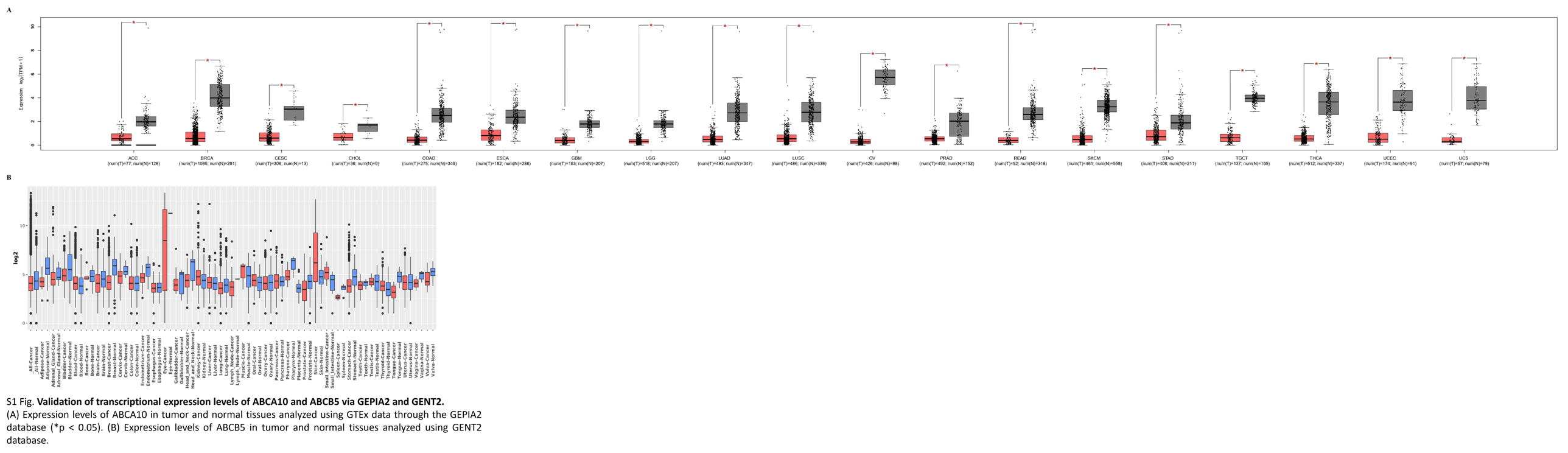

Supplement: S1 Fig [file pone.0308585.s007.tiff]
